# Clinical significance of regions of homozygosity detection in prenatal chromosomal microarray analysis

**DOI:** 10.1016/j.xhgg.2025.100549

**Published:** 2025-11-19

**Authors:** Ying Hao, Qian Geng, Xingping Li, Jingxin Yang, Yang Liu, Qingfa Huang, Yong Xu, Peining Li, Jiansheng Xie, Weiqing Wu, Bo Wu, Wenlan Liu

**Affiliations:** 1 Medical Genetic Center, Shenzhen Maternity and Child Healthcare Hospital, Women and Children’s Medical Center, Southern Medical University, Shenzhen, China; 2 Department of Genetics, Yale University School of Medicine, New Haven, CT, USA; 3 Reproductive Medicine and Prenatal Diagnosis Center, Division of Prenatal Diagnosis, The University of Hong Kong-Shenzhen Hospital, Shenzhen, China; 4 Shenzhen Key Laboratory of Maternal and Child Health and Diseases, Shenzhen, China

**Keywords:** chromosome microarray analysis, copy-number variant, prenatal diagnosis, regions of homozygosity, uniparental disomy, exome sequencing

## Abstract

Chromosomal microarray analysis (CMA) detects pathogenic copy-number variants (pCNVs) and regions of homozygosity (ROHs) in prenatal genetic analysis. This study evaluates the clinical significance of ROH detection in prenatal settings. We reviewed 178 fetuses with ROH detected by CMA among 20,546 fetuses from 2015 to 2023. Clinical and laboratory results, including ultrasound anomalies, cell-free DNA (cfDNA) screening, karyotyping, exome sequencing (ES), and methylation-specific multiplex ligation-dependent probe amplification (MS-MLPA), were analyzed. These 178 fetuses with ROH accounted for 0.87% of prenatal cases. Among them, 24.2% had positive cfDNA screening results, and 52.8% underwent follow-up ES, trio CMA, and MS-MLPA. Follow-up studies detected pathogenic homozygous variants within ROH in two fetuses and uniparental disomy (UPD)-related diseases in five fetuses. Our results and findings from the other five large prenatal case series from literature indicated that ROH detection in prenatal CMA has a baseline positive predictive value of 2.7% for autosomal-recessive disorders, 9.6% for UPD-related diseases, and 0.04% overall additive diagnostic yield. These findings support the use of ES and MS-MLPA for follow-up testing and provide guidance for genetic counseling in fetuses with ROH.

## Teaser

This study evaluates the clinical significance of regions of homozygosity (ROH) detection in prenatal settings. Our results and findings from the other five large prenatal case series from the literature indicated that the use of exome sequencing and methylation-specific multiplex ligation-dependent probe amplification for follow-up testing and provide guidance for genetic counseling in fetuses with ROH.

## Introduction

Regions of homozygosity (ROHs), also known as long contiguous stretches of homozygosity, refer to uninterrupted regions of homozygous alleles with a neutral copy-number state.^1^ ROHs can arise from various mechanisms, including parental relatedness (or consanguinity), chromosomal recombination or rearrangements, trisomy rescue, monosomy rescue, gamete complementation, and postzygotic mitotic nondisjunction.^2^ The presence of ROHs can indicate uniparental disomy (UPD), identity by descent (IBD), and consanguinity based on their distribution and proportion in the genome. Large ROHs observed on a single chromosome may suggest UPD, while IBD typically appears as small (<4 Mb) segments of ROHs in isolated populations without direct parental relatedness.^3^ Consanguinity is suspected when multiple long ROHs are present on several chromosomes.^4^

Chromosomal microarray analysis (CMA) has significantly enhanced the analytical resolution and diagnostic yield for detecting pathogenic copy-number variants (pCNVs) and ROHs in prenatal and pediatric genetic evaluations.^5^ The size and distribution of ROHs vary among normal populations.^6^ In 2013, the American College of Medical Genetics and Genomics (ACMG) developed standards and guidelines for reporting ROHs as incidental findings.^3^ While ROHs themselves are not pathogenic, they can indicate autosomal-recessive (AR) disorders with homozygous pathogenic variants within the ROHs or UPD-related diseases involving imprinted regions.^5^^,^^7^ In 2018, ACMG recommended follow-up studies, including sequencing for AR disorders, microsatellite marker testing, and methylation analysis for UPD, after detecting ROHs.^8^ However, the diagnostic yield and pregnancy outcomes for AR disorders and UPD-related diseases in prenatal fetuses with ROHs remains unclear.

In this study, we reviewed fetuses with ROHs detected by CMA from 2015 to 2023 and collected data from ultrasound examination, cell-free DNA (cfDNA) screening, and follow-up tests, including exome sequencing (ES), trio CMA, and methylation-specific multiplex ligation-dependent probe amplification (MS-MLPA). We identified cytogenomic abnormalities by karyotyping and CMA, pathogenic variants for AR disorders within ROHs by ES, and UPD-related diseases by MS-MLPA and trio CMA. We compared the detection rates of AR disorders and UPD-related diseases with those reported in five other large Chinese prenatal case series. Our findings provide evidence to support timely follow-up genetic analysis and informative genetic counseling for fetuses with ROH.

## Material and methods

### Case series

This retrospective study was approved by the Shenzhen Maternity and Child Healthcare Hospital. Pregnant women who provided informed consent for invasive prenatal diagnosis from April 2015 to December 2023 were included. Fetal samples were collected via chorionic villus sampling (CVS) at 11–14 weeks of gestation (wog), amniocentesis at 17–24 wog, or cordocentesis after 24 wog. Gestational age was confirmed based on the last menstrual period and an ultrasound dating scan at 11–14 wog.

For fetuses with ROH, we collected demographic data, family history, indications for prenatal diagnosis, ultrasonographic findings, maternal serum screening results for aneuploidy, cfDNA screening results, invasive prenatal diagnosis results, pregnancy outcomes, and postnatal follow-up data. All prenatal data and some postnatal follow-up data collected via telephone call were obtained from medical records in the Shenzhen Maternity and Child Healthcare Management Information System.

### Karyotyping and CMA

Cell culture of CVS, amniotic fluid (AF) and umbilical cord blood collection, Giemsa banding, and chromosome analysis were performed as previously described.^9^ Karyotype descriptions were based on the International System for Human Cytogenomic Nomenclature (ISCN 2020).

Genomic DNA was extracted using a QIAamp DNA Blood Mini Kit (Qiagen, Hilden, Germany) from CVS (15 mg), AF (10 mL), or umbilical cord blood (1 mL). All prenatal samples were tested to rule out maternal cell contamination. CNVs and ROH were detected using a high-resolution single-nucleotide polymorphism (SNP) array on a CytoScan 750K platform (Thermo Fisher Scientific, Waltham, MA), which contains 200,000 SNP loci and 550,000 CNV loci for genome-wide coverage. All procedures followed the manufacturers’ protocols. Raw data were analyzed using the Chromosome Analysis Suite with genomic coordinates following the University of California, Santa Cruz genome browser GRCH37/hg19 assembly.

Quality control (QC) criteria included a median absolute pairwise difference ≤0.25, SNP QC ≥15, and a waviness standard deviation ≤0.12. ROH reporting criteria, following ACMG guidelines, were an interstitial ROH >10 Mb, a terminal ROH >5 Mb, and multiple ROHs encompassing >3% of the genome.^3^^,^^10^ The percentage of the genome comprising ROHs was estimated by summing their sizes (>5 Mb) and dividing by the autosomal length (∼2,781 Mb).^3^ Parental blood samples were collected, with informed consent, to verify the origin of fetal ROHs for uniparental isodisomy or heterodisomy (iso/hetero UPD) using CMA and UPDtool.^11^ UPDtool defines isodisomy by >85% homozygous SNPs and heterodisomy by >90% SNPs identical to one parent.

### Follow-up ES and MS-MLPA

DNA samples extracted from fetal specimens and parental peripheral blood specimens were used to construct DNA libraries for ES. Target gene exons and adjacent regions were captured and enriched using the Berry_NanoWESv2 chip and sequenced on the Illumina platform (Illumina, San Diego, CA) to a mean coverage >100×, with >99% of the exome at ≥ 20× coverage. Raw fastq data were analyzed using a pipeline including mapping, realignment, variant calling, QC, variant filtration, and annotation. Variants were classified into five levels according to ACMG standards: pathogenic (P), likely pathogenic (LP), variants of uncertain significance (VUS), likely benign (LB), and benign (B).^12^ P/LP variants were confirmed by Sanger sequencing using an ABI3500Dx Genetic Analyzer (Applied Biosystems, Foster City, CA). P/LP variants within or outside the detected ROH were summarized.

MS-MLPA was used to detect CNVs and methylation status of imprinted loci, following the manufacturer’s manual (MRC Holland, Amsterdam, the Netherlands). The SALSA MS-MLPA kits ME028-D1, ME030-C3, and ME034-C1 were used to detect UPD-related diseases well documented in the Online Mendelian Inheritance in Man (http://www.omim.org), including transient neonatal diabetes mellitus (MIM: 601410), Silver-Russell syndrome (SRS [MIM: 180860]), Beckwith-Wiedemann syndrome (BWS [MIM: 130650]), Temple syndrome (MIM: 616222), Kagami-Ogata syndrome (KOS [MIM: 608149]), Prader-Willi syndrome (PWS [MIM: 176270]), Angelman syndrome (AS [MIM: 105830]), and pseudohypoparathyroidism type 1B (MIM: 603233). Polymerase chain reaction products were analyzed using Coffalyser.Net software (http://www.coffalyser.net) on an ABI Prism 3730 Genetic Analyzer (Applied Biosystems).

### Literature review and inclusion criteria

A PubMed search was conducted using the keywords cfDNA screening, ROH, CMA, prenatal diagnosis, CNV, ES, AR, and UPD. Large prenatal case series studies using CMA for CNV and ROH detection and follow-up genetic analyses for AR disorders and UPD-related diseases were selected and reviewed. Five reports of large prenatal case series of ROHs in the Chinese population, including four single-center studies^7^^,^^13^^,^^14^^,^^15^ and one multi-center study^16^ met the criteria. The percentage of fetuses reported with ROH, the rate of follow-up analyses, the diagnostic findings for AR disorders and UPD-related diseases, and the additive diagnostic yield were summarized.

## Results

### Case series with ROH

In our study, 178 fetuses (0.87%) were identified with ROHs among 20,546 pregnancies undergoing invasive prenatal diagnosis from 2015 to 2023. These fetuses were from 162 AF samples, 9 CVS samples, and 7 umbilical cord blood samples. The pregnancies included 173 singletons and 5 twin pregnancies, with 77 primiparas and 101 multiparas. The maternal age ranged from 21 to 46 years (mean 31 ± 5 years), and the gestational age ranged from 12 to 33 wog (mean 20 ± 5 weeks). The indications for invasive prenatal diagnosis and positive findings in follow-up tests are detailed in Table 1. Ultrasound abnormalities (UAs) were the most common indication (*n* = 82), followed by abnormal cfDNA screening results (*n* = 34). Other indications included high-risk maternal serum screening (*n* = 23), parental genetic factors (*n* = 17), previous adverse pregnancy outcomes (*n* = 11), advanced maternal age (*n* = 7), consanguineous marriage (*n* = 3), and others (*n* = 1). Detailed cfDNA screening results, ultrasound findings, karyotype and CMA results, follow-up ES and MS-MLPA results, and pregnancy outcomes for these 178 fetuses are listed in Table S1.

**Table 1:** Indications for invasive prenatal diagnosis and positive findings by follow-up tests in 178 fetuses with ROH

Indications^a^	Fetuses with ROH	No. of fetuses for follow-up tests	No. of positive findings by follow-up tests
UAs^b^	82	46	4
cfDNA abnormalities	34	15	1
High risk on maternal serum screening	23	12	2
Parental genetic factors	17	8	0
Previous adverse pregnancy outcome	11	7	0
Advanced maternal age	7	4	0
Consanguineous marriage	3	1	0
Others^c^	1	1	0
Total	178	94	7

cfDNA, cell-free DNA; ROH, regions of homozygosity; UAs, ultrasound abnormalities.

a Each case was classified based on its most important indication. The classification of indications, arranged in the order of importance, are as listed in the first column.

b UAs include structural malformations, soft-marker anomalies, fetal growth restriction or overgrowth, and amniotic fluid volume abnormality.

c Others include the normal fetus of twins.

### UAs and cfDNA screening results

As summarized in Table 2, UAs were observed in a total of 82 fetuses. Of these, 30 fetuses (37%) had isolated ultrasound soft-marker anomalies, with increased nuchal translucency (>2.5 mm) being the most common, present in 18 fetuses. Other notable soft-marker anomalies included choroid plexus cyst, widening of the lateral ventricle, and nasal bone absence/shortening, each observed in 8 fetuses. Additionally, 21 fetuses (26%) had multiple malformations, 15 fetuses (18%) had isolated structural malformations involving 7 systems and abdominal wall anomalies, and 14 fetuses (17%) had fetal growth restriction. Amniotic fluid volume abnormality was noted in 2 fetuses (2%).

**Table 2:** Classification of abnormal ultrasound findings in 82 fetuses with ROH

Ultrasound finding	Detailed manifestation	Fetuses with ROH (%)
Isolated ultrasound soft-marker anomaly	Increased NT; nasal bone absence or short, choroid plexus cyst; widening of lateral ventricle; ventricular echogenic spot; tricuspid regurgitation; single umbilical artery; umbilical cord cyst; non-visualization of 12th rib; abnormal course of umbilical artery	30 (36.59)
Multiple malformations		21 (25.61)
Isolated structural malformation		
Central nervous system	ependymal neoplasms, low position of conus medullaris	4 (4.88)
Skeletal system	dysplasia of the foot, syndactyly, limb flexion	2 (2.44)
Cardiovascular system	double aortic arch, ventricular septal defect	2 (2.44)
Digestive system	intestinal duplication	2 (2.44)
Malformations of abdominal wall	omphalocele	2 (2.44)
Stomatognathic system	Ⅲ degree cleft lip with alveolar process cleft	1 (1.22)
Lymphatic system	hygroma colli	1 (1.22)
Urinary system	renal cyst	1 (1.22)
FGR		14 (17.07)
Amniotic fluid volume abnormality	oligohydramnios	2 (2.44)
Total		82 (100)

FGR, fetal growth restriction; NT, nuchal translucency; ROH, regions of homozygosity.

Among the 178 fetuses with ROHs, 43 had positive cfDNA screening results (Table S1). These included high-risk findings for trisomies of chromosomes 21, 18, and 13 in 9 fetuses, duplications of whole or partial chromosomes in 27 fetuses, deletions of whole or partial chromosomes in 6 fetuses, and 1 fetus with a sex chromosome deletion and a duplication in chromosome 16.

### Karyotyping and CMA results

Karyotyping was performed on 148 of the 178 fetuses with ROHs. Among these, 127 yielded normal results, 2 had culture failures, and 19 had abnormal results (Table S1). Notably, CMA identified a Robertsonian translocation 45,XN,rob(14;21) (q10;q10) with UPD14pat in case 10 and an isochromosome 22 as 45,XN,i(22) (q10) by arr(22)×2 hmz in case 56.

**Figure 1:**
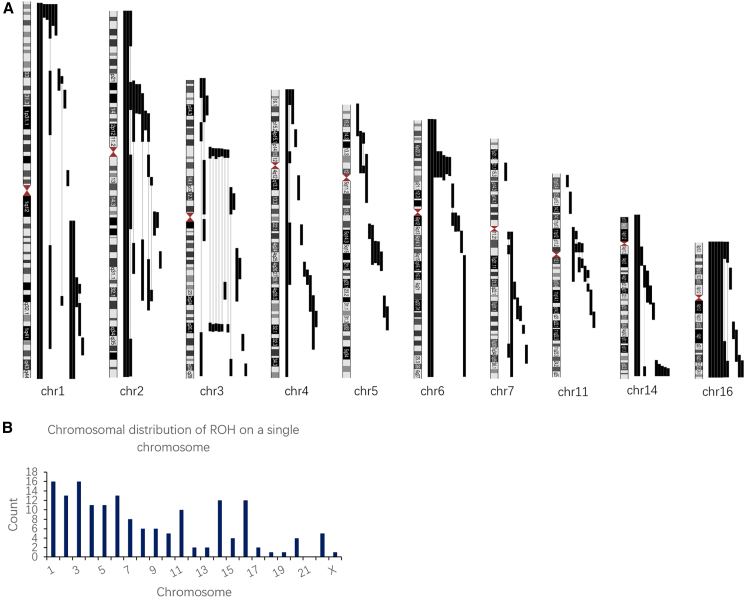
Chromosomal distribution of ROH on a single chromosome (A) Lines to the right of each chromosome represent the location of ROHs carried by each fetus. The length of the lines indicates the range of the corresponding ROHs. ROHs were most frequently distributed on chromosomes 1, 2, 3, 4, 5, 6, 7, 11, 14, and 16. (B) Histograms showing the chromosomal distribution of ROHs on a single chromosome. ROH, regions of homozygosity.

Of the 178 fetuses with ROHs, 168 (94%) had ROHs >10 Mb and 10 (6%) had ROHs ≤10 Mb. For CNVs, 18 fetuses (10%) had pCNVs, 5 fetuses (3%) had CNVs of unknown significance, and 155 fetuses (87%) were normal. Isolated ROHs were found in 161 fetuses (90%), and 2 or more ROHs in multiple chromosomes were observed in 17 fetuses (10%). Among the 161 isolated ROHs in a single chromosome, the most frequently affected chromosomes were chromosomes 1 and 3 (*n* = 16), 2 and 6 (*n* = 13), and 14 and 16 (*n* = 12), followed by chromosomes 4, 5, 11, and 7 (Figure 1A). ROHs were detected in all autosomes except chromosome 21 (Figure 1B). Of these 161 ROHs, 139 were present in partial fragments of chromosomes (ranging from 5.1 to 134 Mb), and 22 covered the entire length of chromosomes 1, 2, 4, 6, 8, 9, 10, 14, 15, 16, 18, 22, and X. Eight fetuses (cases 14, 72, 101, 124, 131, 136, 149, and 158) exhibited mosaic ROHs, with mosaicism proportions ranging from 20% to 80%.

Among the 17 fetuses with multiple ROHs, 3 had ROHs encompassing less than 3% of the genome, while 14 had ROHs covering more than 3% of the genome (ranging from 3% to 9.5%). This suggests a degree of parental relatedness ranging from fourth (3%) to second (12.5%).^1^ Using a reporting cutoff of 3% ROHs in the genome, parental relatedness was suspected in 8% (14/178) of these fetuses. After genetic counseling, 11 cases were confirmed to have consanguineous marriages, 2 cases showed no evidence of parental consanguinity, and 1 case was lost to follow-up. Therefore, the percentage of consanguinity was estimated to be 79% (11/14) in fetuses with ROH >3%. The fetus with the highest ROH percentage (9.5%) was from a first-cousin marriage (case 38). Two consanguineous couples (cases 73 and 87) had previously experienced adverse pregnancy outcomes associated with an AR disorder.

**Figure 2:**
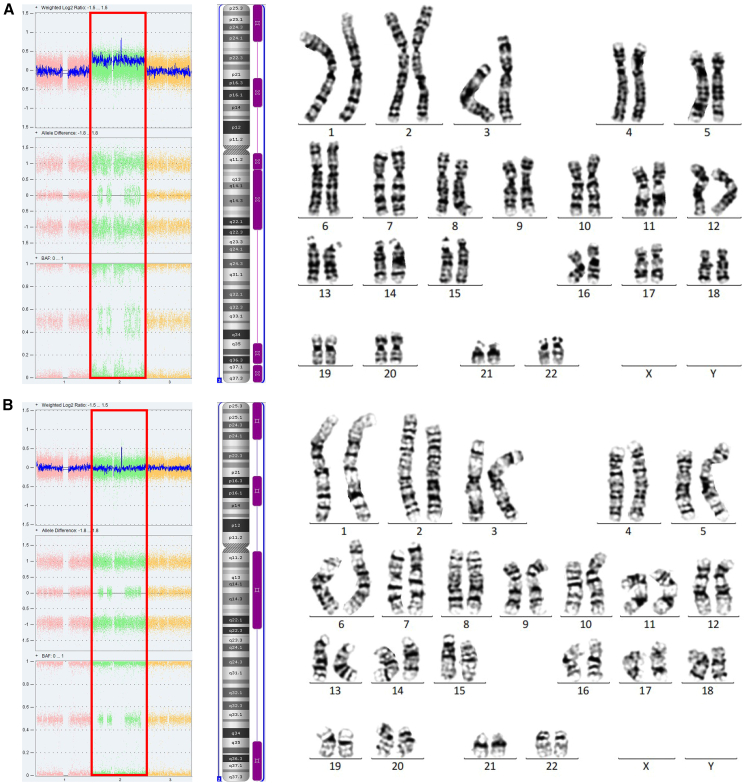
CMA result and karyotyping of case 15 (A) AF: WGV shows 40% mosaicism for trisomy 2 in uncultured AF. AD and BAF show ROH (isodisomy area) in chromosome 2. The smooth signal of chromosome 2 is 2.4 (red box) (left). Karyoview shows four large ROH fragments (purple bars) in uncultured AF (center). Karyotyping of cultured AF shows 46, XN,1qh+,16qh+ (right). (B) Umbilical cord blood: WGV shows no trisomy 2. AD and BAF show ROH (isodisomy area) in chromosome 2. The smooth signal of chromosome 2 is 2 (red box) (left). Karyoview shows four large ROH fragments (purple bars) (center). Karyotyping shows 46, XN,1qh+,16qh+ (right). AD, allele difference; AF, amniotic fluid; BAF, B allele frequency; CMA, chromosomal microarray analysis; ROH, regions of homozygosity; WGV, whole-genome view.

Notably, in case 15, CMA detected 40% mosaicism for trisomy 2 with 4 large ROHs in uncultured AF: 2p25.3p24.1 (23.66 Mb), 2p21p14 (18.54 Mb), 2q11.1q22.3 (49.46 Mb), and 2q35q37.3 (24.94 Mb) (Figure 2A, left and center panels). However, only a disomic pattern and the four large ROHs at 2p and 2q were identified in umbilical cord blood (Figure 2B, left and center panels). Trisomy 2 was not observed in the karyotype of cultured AF or umbilical cord blood (Figures 2A and 2B, right panel). These results indicated a rescue of trisomy 2 to disomy 2, with retention of segmental isodisomy from one parent. The fetus was born at full term with a birth weight of 1,900 g. Follow-up at 6 years and 9 months showed that the child’s growth parameters were within the normal range.

### Follow-up ES results

**Figure 3:**
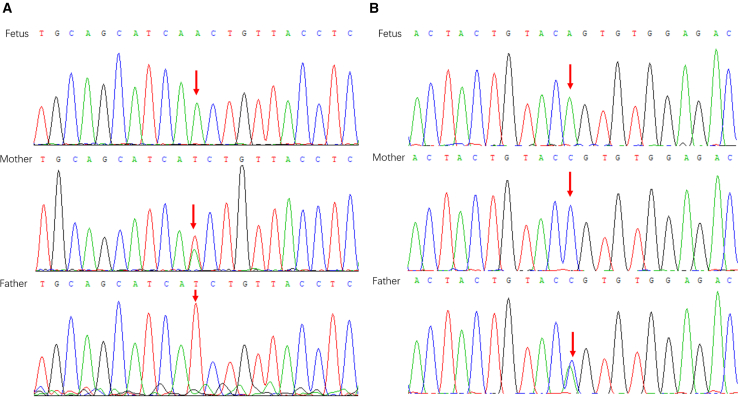
Sanger sequencing verifies variants detected by trio ES in cases 75 and 152 (A) Case 75: the fetus has a homozygous variant, NM_000500.7:c.518T>A (p.Ile173Asn), in the CYP21A2 gene on chromosome 6 within the detected ROH. Red arrows indicate the homozygous A in the fetus, heterozygous AT in the mother, and homozygous T in the father, respectively. (B) Case 152: the fetus has a homozygous variant, NM_017739.4:c.1324C>A (p.Arg442Ser), in the POMGNT1 gene on chromosome 1 within the detected ROH. Red arrows indicate the homozygous A in the fetus, homozygous C in the mother, and heterozygous AC in the father, respectively. ES, exome sequencing.

ES or trio ES was performed on 60 of the 178 fetuses (33.7%). Of these, 45 fetuses had normal results, 1 fetus had simple trisomy X, and 14 fetuses were detected with variants, among whom 7 fetuses carried 9 homozygous variants: 3 P/LP variants and 6 VUS variants (Table 3). The parents of the seven fetuses carrying homozygous variants were not consanguineous. Diagnosis of AR disorders were provided in two fetuses with homozygous P/LP variants within the ROH (cases 75 and 152). Case 75 showed a 38.482-Mb ROH on 6p25.3p21.2 containing a maternal *CYP21A2* (MIM: 613815) variant, NM_000500.7:c.518T>A (p.Ile173Asn), causing congenital adrenal hyperplasia due to 21-hydroxylase deficiency (21-OHD-CAH [MIM: 201910]) (Figure 3A). The couple elected termination of pregnancy (TOP) at 34 wog after genetic counseling. Case 152 exhibited arr(X)x3,(1)x2 hmz with a paternal *POMGNT1* (MIM: 606822) variant NM_017739.4:c.1324C>A (p.Arg442Ser), associated with muscular dystrophy-dystroglycanopathy type A3 and type B3 (MDDGA3/B3 [MIM 253280/613151]) (Figure 3B). The couple elected TOP at 29 wog.

**Table 3:** ES findings in seven fetuses with nine homozygous variants

Case	Disorders	ES finding (hg19)	Zygosity	Inheritance	Inheritance patterns	ACMG classification
40	NA	*EEF1D*: NM_032378.4:c.1114delC (p.Leu372Ter) chr8:144669000	homozygous	mat/pat	NA	VUS
	epidermolysis bullosa simplex, with pyloric atresia; muscular dystrophy, limb-girdle, type 2Q; epidermolysis bullosa simplex, Ogna type; epidermolysis bullosa simplex, with muscular dystrophy	*PLEC*: NM_000445.3:c.4327C>T (p.Arg1443Cys) chr8:144999851	homozygous	mat/pat	AR/AD	VUS
50	psoriasis 14, pustular	*IL36RN*: NM_173170.1:c.152A>T (p.Asp51Val) chr2:113819737	homozygous	pat	AR	VUS
58	malignant hyperthermia susceptibility 5; hypokalemic periodic paralysis, type 1, HOKPP1; thyrotoxic periodic paralysis, susceptibility to, 1 (TTPP1)	*CACNA1S*: NM_000069.2:c.3364T>C (p.Tyr1122His) chr1:201029836	homozygous	NA	AD	VUS
75	adrenal hyperplasia, congenital, due to 21-hydroxylase deficiency	*CYP21A2*: NM_000500.7:c.518T>A (p.Ile173Asn) chr6:32007203	homozygous	mat	AR	P
84	myasthenic syndrome, congenital, 8	*AGRN*: NM_198576.3:c.1907G>A (p.Gly636Asp) chr1:979311	homozygous	NA	AR	VUS
	Joubert syndrome 25	*CEP104*: NM_014704.3:c.896G>A (p.Arg299Gln) chr1:3754079	homozygous	NA	AR	VUS
152	muscular dystrophy-dystroglycanopathy (congenital with brain and eye anomalies), type A, 3; muscular dystrophy-dystroglycanopathy (congenital with impaired intellectual development), type B, 3	*POMGNT1*: NM_017739.4:c.1324C>A (p.Arg442Ser) chr1:46658069	homozygous	pat	AR	LP
159	microcephaly 5, primary, autosomal recessive	*ASPM*: NM_018136.5:c.8214dup (p.Gln2739SerfsTer12) chr1:197070166	homozygous	mat/pat	AR	P

ACMG, American College of Medical Genetics and Genomics; AD, autosomal dominant; AR, autosomal recessive; ES, exome sequencing; LP, likely pathogenic; mat, maternal; NA, not available; P, pathogenic; pat, paternal; VUS, variants of uncertain significance.

### Follow-up trio CMA and MS-MLPA results

**Figure 4:**
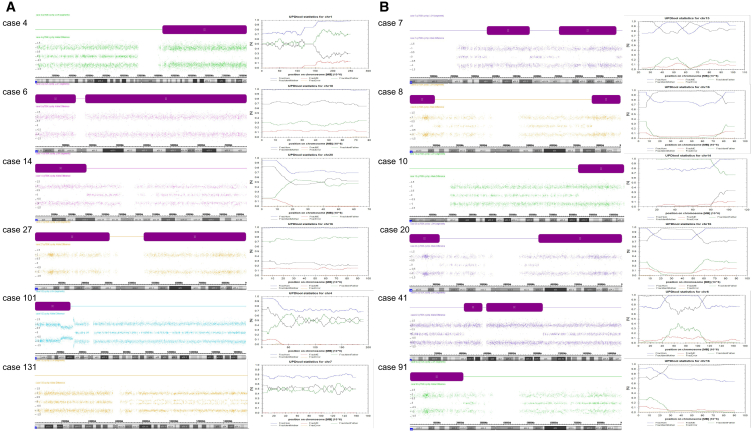
Twelve fetuses of ROH further confirmed as UPD by trio CMA Left: detection of segmental or whole ROHs (purple bars) on one chromosome by CMA. AD indicates the genotype of each SNP. For non-ROH, there are three SNP combinations, AA, AB, and BB, presenting as three strips on the graph. For ROH, there are two SNP combinations, AA and BB, presenting as two strips on the graph. Right: confirmation of UPD by UPDtool of trio CMA. FracHom (blue line): fraction of genotypes that are homozygous; FracME (red line): fraction of Mendelian errors; FracFather (green line): fraction of genotypes identical to the father; FracMother (black line): fraction of genotypes identical to the mother; FracError (orange line): fraction of genotyping errors (errors that cannot be explained by UPD). (A) Cases 4, 6, 14, 27, 101, and 131 were isodisomy of segmental or whole ROHs. (B) Cases 7, 8, 10, 20, 41, and 91 were mixed iso/hetero UPD of whole chromosome. UPD, uniparental disomy.

Trio CMA was performed on 33 of the 178 fetuses (18.5%). UPDtool analysis demonstrated UPD in 12 fetuses and IBD in 21 fetuses. Isodisomy was identified in chromosomal segments or whole chromosomes in six fetuses (cases 4, 6, 14, 27, 101, and 131) (Figure 4A). Mixed iso/hetero UPD of whole chromosomes were found in 6 fetuses (cases 7, 8, 10, 20, 41, and 91) (Figure 4B). Three fetuses (cases 7, 10, and 131) were diagnosed with UPD-related diseases.

MS-MLPA was performed on 27 fetuses (15.2%), and normal copy number ratios were detected in all but 6 fetuses with abnormal methylation status (cases 41, 44, 72, 82, 172, and 176).

**Figure 5:**
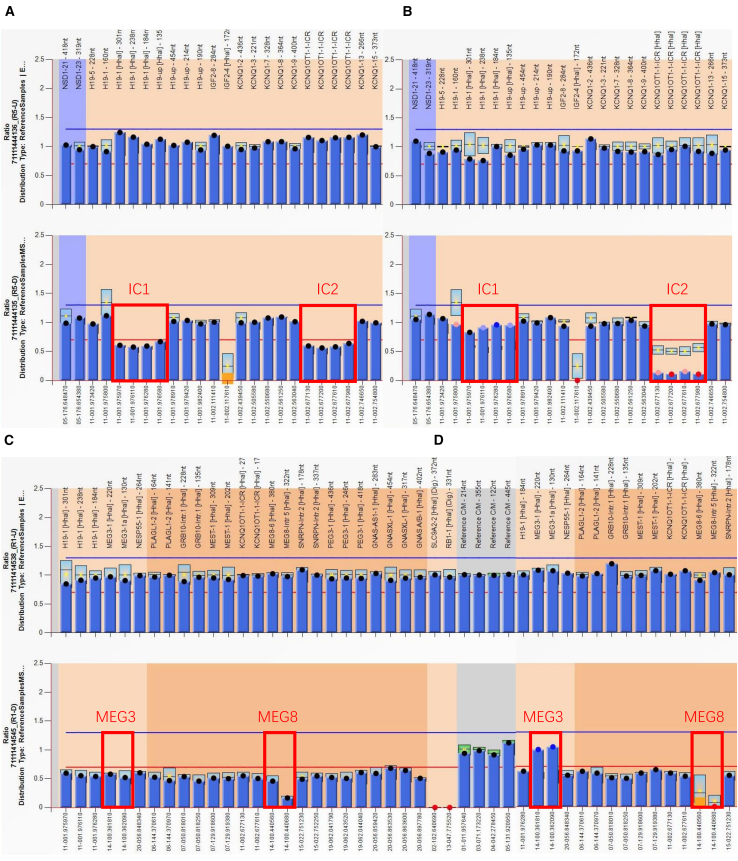
MS-MLPA results of cases 72 and 82 Electropherograms and normalized data for ME030-C3 (A) normal control; (B) case 72 and ME034-C1; (C) normal control; and (D) case 82. Both fetuses had a normal copy-number ratio of 1 (B and D, top). Case 72: gain of methylation ratio of IC1 (H19DMR of paternal methylation) and loss of methylation ratio of IC2 (KvDMR [Kv differentially methylated region] of maternal methylation) (B, bottom), in comparison with 0.5 from a normal control (A, bottom) (red box). Case 82: methylation ratio of MEG3 (paternal methylation) was 1 and methylation ratio of MEG8 (maternal methylation) was 0 (D, bottom), in comparison with 0.5 from a normal control (C, bottom) (red box). IC1, imprinting center 1; IC2, imprinting center 2; MS-MLPA, methylation-specific multiplex ligation-dependent probe amplification.

Three fetuses (cases 72, 82, and 172) were associated with UPD-related diseases. Of these, 2 fetuses (cases 72 and 82) were identified in the context of ROH on the relevant chromosome and included the critical region of the UPD-related diseases. The methylation ratio indicated UPD11p15.5p15.4pat for BWS (Figures 5A and 5B) and UPD14pat for KOS (Figures 5C and 5D) in cases 72 and 82, respectively.

In total, five fetuses were diagnosed with UPD-related diseases via CMA and follow-up MS-MLPA/trio CMA: 1 maternal UPD15 (PWS) continuing to term with postnatal clubfoot and mild obesity (case 7), 2 paternal UPD14 (KOS) terminated at 25–27 wog (cases 10 and 82), 1 80% mosaic paternal UPD11p15 (BWS) terminated at 26 wog (case 72), and 1 20% mosaic maternal UPD7q (SRS) terminated at 26 wog (case 131). Ultrasound findings matched expected phenotypes in all terminated fetuses.

### Pregnancy outcomes and postnatal follow-up

The pregnancy outcomes for the 178 fetuses with ROH included 110 term births, 41 fetuses of TOP, 19 preterm births (one of which resulted in neonatal death), 3 intrauterine deaths, 2 miscarriages, and 3 fetuses that were lost to follow-up. Among these, a premature infant (case 54) was diagnosed with bilateral subependymal cyst, persistent left superior vena cava, congenital atrial septal defect, hyperlactacemia, and type II oxidative phosphorylation deficiency. This infant also experienced life-threatening neonatal metabolic acidosis with agenesis of the corpus callosum 2 months after birth.

In full-term fetuses, case 57 was found to have third-degree cleft lip and palate both prenatally and postnatally, and underwent surgery at 4 months of age. Case 61 was prenatally diagnosed with left kidney absence or severe dysplasia and a ventricular septal defect. Postnatally, additional abnormalities including double uterus, double vagina, and double cervix were identified. Follow-up at 5 years of age revealed that the ventricular septal defect had healed on its own, kidney function was normal upon annual review, and the structural abnormalities of the female reproductive organs did not require intervention at the time. Case 86, who had omphalocele (mostly involving the liver), underwent surgery after full-term delivery and is now 4 years old and recovering well. Case 127, diagnosed with double aortic arch, had heart surgery 1 year after birth and is now 2 years old and healthy.

Overall, the study showed that the rate of adverse perinatal outcome (APO), including TOP, neonatal death, fetal intrauterine death, and miscarriage, was 26.9% (47/175). The APO rate of 34.6% (28/81) in ROH fetuses with UAs was higher than the 20.2% (19/94) in those without UAs.

### Results from literature review

Data from our study and five other large prenatal case series^7^^,^^13^^,^^14^^,^^15^^,^^16^ reported in the literature are summarized in Table 4. These studies investigated AR disorders and UPD-related diseases underlying ROH detected by CMA. The fetuses in these studies came from prenatal diagnosis centers, and the average detection rate of ROH was 0.85%. The proportion of ROH in a single chromosome was 83.2% and in multiple chromosomes was 16.8%. The rate for follow-up studies by ES, trio CMA, and MS-MLPA was 37.8%. The detection rates of UPD-related diseases, AR disorders, and additive diagnostic yield were 9.6%, 2.7%, and 0.04%, respectively.

**Table 4:** Summary of follow-up rate, detection rate of UPD-related diseases, AR disorders, and additive diagnostic yield

Study	Sample size	Array platform	Detection rate of ROH	ROH in a single chromosome	Multiple ROH	Rate of follow-up studies	Follow-up methods
Our study	20,546	CytoScan 750K	0.87% (178/20,546)	90.4% (161/178)	9.6% (17/178)	52.8% (94/178)	ES, trio CMA, MS-MLPA
Liu et al.^14^	10,294	CytoScan HD	0.97% (100/10,294)	81.0% (81/100)	19.0% (19/100)	25.0% (25/100)	ES, trio CMA
Liang et al.^7^	5,063	CytoScan 750K	0.43% (22/5,063)	77.3% (17/22)	22.7% (5/22)	50.0% (11/22)	ES, trio CMA
Zhu et al.^13^	5,116	CytoScan 750K/300K	0.76% (39/5,116)	64.1% (25/39)	35.9% (14/39)	10.3% (4/39)	ES
Xue et al.^16^	11,062	CytoScan 750K	0.96% (106/11,062)	83.0% (88/106)	17.0% (18/106)	39.6% (42/106)	ES, trio CMA
Ma et al.^15^	6,288	CytoScan 750K/HD	0.80% (50/6,288)	80.0% (40/50)	20.0% (10/50)	22.0% (11/50)	ES, trio CMA, MS-MLPA
Total	58,369	–	0.85% (495/58,369)	83.2% (412/495)	16.8% (83/495)	37.8% (187/495)	–

Detection rate of UPD-related diseases	Additive diagnostic yield of UPD-related diseases	Detection rate of AR disorders	Additive diagnostic yield of AR diseases	Total additive diagnostic yield
5.3% (5/94)	0.02% (5/20,546)	2.1% (2/94)	0.01% (2/20,546)	0.03% (7/20,546)
4.0% (1/25)	0.01% (1/10,294)	0 (0/25)	0 (0/10,294)	0.01% (1/10,294)
18.2% (2/11)	0.04% (2/5,063)	0 (0/11)	0 (0/5,063)	0.04% (2/5,063)
0 (0/4)	0 (0/5,116)	25.0% (1/4)	0.02% (1/5,116)	0.02% (1/5,116)
23.8% (10/42)	0.09% (10/11,062)	4.8% (2/42)	0.02% (2/11,062)	0.11% (12/11,062)
0 (0/11)	0 (0/6,288)	0 (0/11)	0 (0/6,288)	0 (0/6,288)
9.6% (18/187)	0.03% (18/58,369)	2.7% (5/187)	0.01% (5/58,369)	0.04% (23/58,369)

AR, autosomal recessive; CMA, chromosomal microarray analysis; ES, exome sequencing; MS-MLPA, methylation-specific multiplex ligation-dependent probe amplification; ROH, regions of homozygosity; UPD, uniparental disomy.

## Discussion

In this study, we retrospectively analyzed 178 fetuses with ROHs to present the ultrasound findings and diagnostic results on cytogenomic abnormalities, AR disorders, and UPD-related diseases. The detection rate of ROHs in our case series was 0.87%, which is consistent with the detection rate of 0.43%–0.97% reported from 5 other large prenatal case series in the Chinese population.^7^^,^^13^^,^^14^^,^^15^^,^^16^ The variations in the detection rate could be partially due to the inconsistent reporting criteria of ROHs in different laboratories. In 2021, the ACMG technical standard on ROH interpretation and reporting recommended that the presence of an ROH on a single entire chromosome (iso/hetero) and a single large segment (≥20 Mb) or multiple ROHs in segments of a single and several chromosomes should be reported in prenatal CMA.^17^ In 2023, the guideline^18^ for the application of CMA in prenatal diagnosis in China was released, and the practice of ROH reporting in different laboratories has become more consistent and standardized.

CMA provided accurate detection of CNVs and ROHs to define genomic imbalance and UPD from chromosomal abnormalities. As shown in cases 10 and 56, an UPD14pat for KOS and isodisomy of chromosome 22 were defined from an otherwise Robertsonian translocation. Furthermore, trisomy rescue with resultant UPD was detected in case 15. This integrated cytogenomic interpretation not only provides a clear disease-causing explanation and parental origin of chromosome abnormalities for genetic counseling but it also leads to further investigation into the underlying molecular mechanisms causing trisomy rescue during embryogenesis and fetal development.

The report of ROHs has led to follow-up ES, trio CMA, and MS-MLPA to detect AR disorders and UPD-related diseases. Multiple ROHs in >3% of the genome revealed consanguineous marriages in 11 of 14 fetuses (79%), which highlights the need for attention to familial recurrence of AR disorders, as shown in cases 73 and 87. From 60 follow-up ES, 2 AR disorders within the ROHs were detected: 1 maternal homozygous P variant (case 75) and 1 paternal homozygous LP variant (case 152). Clinically significant imprinting diseases have been reported previously in chromosomes 6, 7, 11, 14, 15, and 20.^1^^,^^2^ In addition, in non-imprinting diseases, UPD 16 shows variable outcomes from normal growth to growth failure.^19^ ROHs detected in these chromosomes should be analyzed further for UPD-related diseases. Follow-up trio CMA and MS-MLPA detected five UPD-related diseases, including UPD7qmat for SRS, UPD11p15pat for BWS, UPD14pat for KOS, and UPD15mat for PWS. The estimated prevalence for well-recognized PWS/AS is 1 in 10,000–30,000 live births, for BWS it is 1 in 10,000–15,000 live births, and the less frequently seen SRS is 1 in 75,000–100,000 live births. In this prenatal case series of 20,546 fetuses, we detected 1 PWS (case 7), 1 BWS (case 72), and 1 SRS (case 131), indicating an effective approach of CMA followed MS-MLPA to detect these rare genetic diseases. Because of the requirement for follow-up ES, trio CMA, and MS-MLPA analyses on fetuses with ROHs, timely genetic counseling and willingness for further study have been the limiting factors in prenatal diagnosis.

Most of these UPD-related diseases occur sporadically due to a range of causative molecular alterations, and prenatal features from ultrasound exams are non-specific. Therefore, genetic counseling is challenging in predicting the clinical outcome of a pregnancy and estimating the recurrence risk for the parents. Prenatal ultrasound screening in fetuses with ROHs demonstrated variable ultrasound presentations but failed to detect common findings associated with specific ROHs. We found that 46% (82/178) of ROH fetuses had UAs, with the most common findings being isolated ultrasound soft-marker anomaly (37%, 30/82) followed by multiple malformations (26%, 21/82). The rate of UAs ranged from 31% to 73% in 5 other prenatal case series.^7^^,^^13^^,^^14^^,^^15^^,^^16^ The most common findings were isolated structural malformation (42%) and multiple malformations (26%) in Liu et al.,^14^ ultrasound soft-marker anomaly (36%) and structural abnormalities (33%) in Xue et al.,^16^ and soft-marker anomaly (24%) in Ma et al.^15^ In summary, more than half of the fetuses with ROHs had UAs, but the proportion of UAs varied among different studies. These differences further illustrated the absence of a correlation between ROHs and specific ultrasound findings, except for some UPD-related diseases with indications for prenatal ultrasound manifestations, such as SRS and BWS.^20^^,^^21^

The rate of APO was 1.7-fold higher in ROH fetuses with UAs (34.6%) than in those without UAs (20.2%), indicating that fetal ultrasound findings provide important information for predicting clinical outcomes. Therefore, carefully examining the ultrasound findings with correlated features of AR disorders and UPD-related diseases could support precise laboratory diagnosis and informative genetic counseling.

The detection rate of AR disorders in fetuses that underwent follow-up analysis was 2.7% (5/187), and the estimated additive diagnostic yield was 0.01% (5/58,369) from our study and the reviewed large prenatal case series.^7^^,^^13^^,^^14^^,^^15^^,^^16^ The additive diagnostic yield in pediatric patients was 0.15% (3/2,050) and 0.18% (4/2,226) in Wen et al.^5^ and Fan et al.,^22^ respectively. The rate was significantly higher in pediatric patients than in prenatal case series, mainly because the phenotypes of pediatric patients were more defined, resulting in a higher detection rate of AR disorders. The detection rate of UPD-related diseases in fetuses that underwent follow-up analysis was 9.6% (18/187), and the estimated additive diagnostic yield was 0.03% (18/58,369) from our study and the reviewed large prenatal case series,^7^^,^^13^^,^^14^^,^^15^^,^^16^ which is similar to an estimated occurrence of UPD-related diseases in 0.05% (1/2,050) of pediatric patients from one case series.^5^

### Conclusions

Results from our study and five other large case series summarized the diagnostic yields of AR disorders and UPD-related diseases in fetuses with ROHs. CMA detected ROHs in approximately 1% of prenatal fetuses. Nearly half (46%) of the fetuses with ROHs had UAs, but there was no correlation between ROHs with specific ultrasound findings. The average detection rates of 2.7% for AR disorders and 9.6% for UPD-related diseases in fetuses with ROHs contributed to additional diagnostic yields of 0.01% and 0.03% in prenatal fetuses, respectively. The APO was 1.7-fold higher in ROH fetuses with UAs than in those without UAs. Detailed ultrasound examination, standardized ROH reporting and interpretation, timely follow-up testing, and informative genetic counseling could improve the detection rates and pregnancy outcomes for AR disorders and UPD-related diseases in prenatal diagnosis.

## Data and code availability

All data supporting this study are included in the article and its supplemental information.

## Acknowledgments

This work was supported by grants from the Shenzhen Health Economics Association (202330), the 10.13039/501100021171GuangDong Basic and Applied Basic Research Foundation (2022A1515220109), the 10.13039/501100012151Sanming Project of Medicine in Shenzhen Municipality (SZSM202311005), and the Shenzhen Key Laboratory of Maternal and Child Health and Diseases (ZDSYS20230626091559006). The authors thank Caiqun Luo, Hui Wang, Liyuan Chen, Xiaoxia Wu, Hu Zhang, and Zhiyong Xu for their help in collecting clinical data for the article.

## Author contributions

W.L. and B.W. supervised the project. J.X. collected the clinical data and provided genetic counseling to pregnant women. Y.H., Q.G., X.L., J.Y., Y.L., Q.H., and Y.X. performed genetic testing and analysis. Y.H. wrote the manuscript. P.L. and W.L. conceived the study, designed the research program, and revised the manuscript. All authors reviewed the manuscript.

## Declaration of interests

The authors declare no competing interests.

## Supplemental information

Table S1. ROH detected in 178 fetuses.
